# Editorial: The impact of sedentary behavior and virtual lifestyles on physical and mental wellbeing

**DOI:** 10.3389/fpubh.2023.1255642

**Published:** 2023-07-21

**Authors:** Fahad Hanna, Emily You, Mohamed El-Sherif

**Affiliations:** ^1^Public Health Program, Torrens University Australia, Melbourne, VIC, Australia; ^2^Academic Unit for Psychiatry of Old Age, Department of Psychiatry, The University of Melbourne, Melbourne, VIC, Australia; ^3^Department of Bariatric Surgery, Hamad Medical Centre, Doha, Qatar

**Keywords:** sedentary behavior, physical activity, COVID-19, lockdown, healthy aging, university students

The purpose of the Research Topic under the umbrella of Public Health Education and Promotion was to encourage and invite submissions aimed at investigating the impact of sedentary behavior and virtual lifestyle on physical and mental health and wellbeing. Research pertinent to the above topic/ theme has been submitted. The topics collectively aimed to raise awareness about sedentary lifestyle/ behavior and its negative consequences, and encouraged people to sit less and move more.

Almost all studies addressed issues around knowledge, awareness and uptake or maintenance of physical activity (PA) and/or sedentary behavior, and the impact of physical inactivity or sedentary behavior on health and health behavior. For example, the qualitative study by You et al. compared perceived benefits of and barriers and enablers to PA in older Caucasian and Chinese adults living in Australia. This study found that regular PA was beneficial to physical, mental and cognitive health, and recommended that community, health care system and governments all have an important role in promoting the uptake and maintenance of PA in the older population (You et al.). A second study (Chong et al.) included older population with mild cognitive impairment or subjective cognitive concerns and analyzed the support person's preferences and perspectives of physical activity programs for this population. This study found that there was a potential benefit in engaging support persons when offering physical activity interventions to older people with mild cognitive impairment or subjective cognitive concerns. The above study added that it would be critical to explore barriers and enablers of physical activity together with translational research around dissemination and implementation of guidelines of physical activity, including behavior change interventions to increase motivation and adherence.

In relation to assessing the impact of inactivity on younger populations, a systematic review by Li et al. tested if sedentary behavior is associated with executive function in children and adolescents. The study assessed the relationship between prolonged sedentary behavior and cognitive functioning and found no reliable evidence of the association between sedentary behavior and executive function in children and adolescents. However, the study found a link between screen time and decline in executive function in these groups. Given that a great percentage of sedentary time may involve “screen time”, these findings are potentially relevant to inactivity in this population.

A cohort study by Zhu et al. investigated the “*Combined effects of PA and sedentary behavior on all-cause mortality in heart failure patients*”. The study analyzed the national health and nutrition examination survey data and found that PA has a protective effect on heart failure patients' prognosis, particularly those with high sedentary behavior. The study also found that sedentary behavior independently exhibited a negative association with improvement in populations without PA.

The study by Loef et al. explored the mediating role of PA and sedentary behavior in the relationship between “working from home and musculoskeletal pain during the COVID-19 pandemic”. The study specifically addressed the impact of the pandemic and working from home on musculoskeletal pain. It found that working from home was a marked risk factor for upper musculoskeletal pain which was not resolved by physical activity. The study recommended a reduction in sedentary behavior to reduce musculoskeletal pain. This is yet another reminder that the negative health impact of prolonged sedentary time is not necessarily corrected by PA.

Research around the impact of COVID-19 pandemic on increased level of sedentary behavior was also explored in this issue. Heller et al. conducted a survey relating to sedentary behavior in university students before and during the COVID-19 pandemic. The survey investigated sedentary time and focused on the level of risk of sedentary behavior using pre-pandemic predictors. It found that university students who were physically active prior to the pandemic had less sedentary time during the pandemic, compared to those with lower or no physical activity. This is a reminder that physical activity in young adults such as university students is a protective factor against sedentary behavior during any potential scenarios of pandemic and other forms of lockdowns.

Our Research Topic has attracted research studies investigating the impact of physical activity and sedentary behavior on health and wellbeing. These studies identified the risk of sedentary behavior for poor or declining health and wellbeing, regardless of age, ethnicity or the level of education. The above studies have consistently recommended that individuals, regardless of their health status, commit to regular physical activity and lower sedentary time to reduce health risk. Governments, policy makers, health care providers, and health program designers should consider the effect of physical activity, or the lack thereof, on health and wellbeing of the population. While this is particularly important for vulnerable populations and those at risk, reduction of sedentary behavior and adoption of physical activity can protect us all from ill health.

## Author contributions

All authors listed have made a substantial, direct, and intellectual contribution to the work and approved it for publication.

